# Boosting efficiency of eco-friendly perovskite solar cell through optimization of novel charge transport layers

**DOI:** 10.1098/rsos.230331

**Published:** 2023-06-07

**Authors:** Muhammad Ismail, Muhammad Noman, Shayan Tariq Jan, Muhammad Imran

**Affiliations:** ^1^ U.S.-Pakistan Center for Advanced Studies in Energy, University of Engineering and Technology, Peshawar, Pakistan; ^2^ National University of Sciences and Technology (NUST) Rawalpindi, Military College of Signals, Pakistan; ^3^ Department of Energy Engineering Technology, University of Technology, Nowshera, Pakistan

**Keywords:** perovskite solar cell, FASnI_3_, carbon ETL, copper HTL, scaps-1D

## Abstract

Formamidinium tin triiodide (FASnI_3_) is a suitable candidate for the absorber layer in perovskite solar cells (PSC) because of its non-toxicity, narrow band gap, thermal stability and high carrier mobility. This study focuses on the analysis and improvement in the performance of FASnI_3_-based PSCs using various inorganic charge transport materials. The copper-based materials such as Cu_2_O, CuAlO_2_, CuSCN and CuSbS_2_ are introduced as hole transport layers due to their earth abundance, ease of manufacturing, high charge mobilities and chemical stability. Similarly, fullerene derivates (PCBM and C_60_) are deployed as electron transport layers due to their mechanical strength, thermal conductivity and stability. The effect of these materials on optical absorption, quantum efficiency, energy band alignment, band offsets, electric field and recombination are studied in detail. The reasons for the low performance of the cell are identified and improved through design optimization. The PSC performance is analysed in both inverted and conventional architecture. Among all the structures, the best result is achieved through ITO/CuSCN/FASnI_3_/C_60_/Al with an efficiency of 27.26%, *V*_oc_ of 1.08 V, *J*_sc_ of 29.5 mAcm^−2^ and FF of 85.6%.

## 1. Introduction

**Figure 1.**
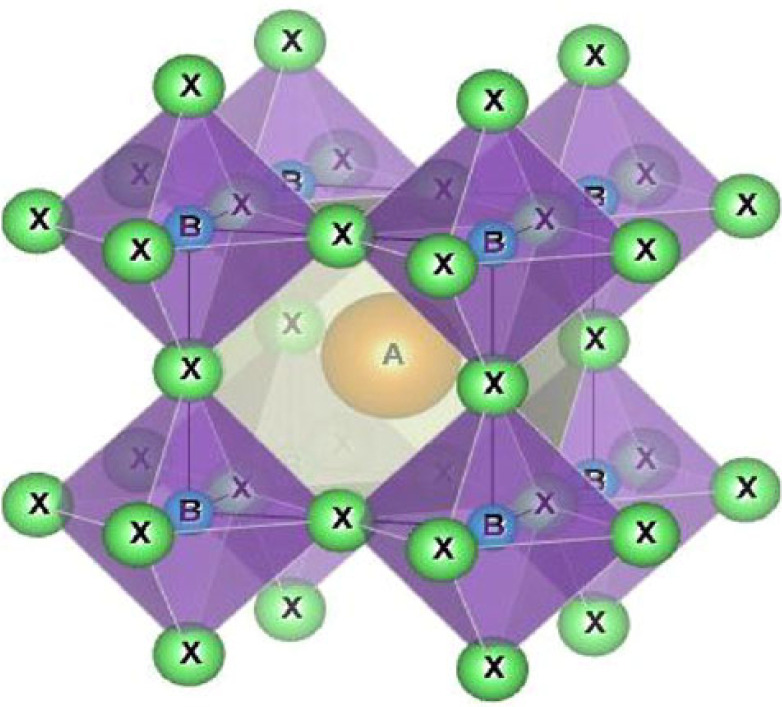
Perovskite ABX_3_ crystal structure.

Over the last decade, perovskite solar cells (PSC) have shown exponential progress in terms of efficiency but structural stability and degradation still remain an obstacle [1]. Perovskites are direct band gap materials with the ABX_3_ crystal structure as shown in figure 1, where ‘A’ is a monovalent organic cation (Formamidinium, Methylammonium, Cesium, etc.), ‘B’ is a divalent metal cation (Sn^+2^, Pb^+2^) and ‘X’ is the halogen anion [2,3]. Perovskites are known for their impressive light harvesting properties because of their high absorption coefficient, large diffusion lengths (0.1–1 µm), tunable band gaps and high carrier mobility (1–10 cm^2^ V^− 1^ s^−1^) [4].

Lead-based PSC have achieved a breakthrough efficiency of 25.7% but the toxic nature of lead limits its large-scale commercialization [5,6]. To address that problem the toxic lead can be replaced with alternative materials such as Sn, Ge, Bi, Sb and Cu [7]. Sn is considered an adequate replacement for the lead as it is non-toxic, lies in the same group as Pb, and shows higher mobilities (10^2^–10^3^ cm^2^ V^−1^ s^−1^) than Pb (10–10^2^ cm^2^ V^−1^ s^−1^) based perovskites [8].

MASnI_3_ and FASnI_3_ are among the high-performing Sn-based perovskites. MASnI_3_ has a narrower bandgap (1.25 eV) when compared with FASnI_3_ (1.41 eV), however, it shows less thermal stability [9]. In Sn-based PSC when FA ($\mathrm{HC}{\left({\mathrm{NH}}_{2}\right)}_{2}^{+}$) cation is used instead of MA (${\mathrm{CH}}_{3}{\mathrm{NH}}_{3}^{+}$) the temperature stability increases which implies the strong hydrogen bonding between organic HC(NH_2_)_2_ and the inorganic matrix [10,11]. FASnI_3_ shows a higher tolerance factor (because of the large ionic radius of FA cation), higher carrier mobility and low auger recombination when compared with MASnI_3_ [12,13]. Recent developments in two-dimensional Ruddlesden popper PSC shows that incorporating β-flouroethamamine cations instead of phenyl ethyl ammonium in FA-based PSCs can improve their performance and efficiency up to 16.77% [14]. The Sn-based solar cells show instability when exposed to air which is attributed to the oxidation of Sn^+2^ to Sn^+4^. Sn^+4^ is not toxic but it behaves like a P-type impurity which consequently decreases PCE (Power Conversion Efficiency) [15]. In FASnI_3_-based PSC, the extent to which Sn^+2^ converts to Sn^+4^ is less compared with MASnI_3_-based solar cells. SnX_2_-based materials are used to tackle this problem [16]. The research also shows that the stability issue regarding the ambient condition can be resolved by incorporating isovalent cations into Perovksite lattice [17]. Until now the highest efficiency reported for FASnI_3_-based solar cells is 14.6% with PEDOT: PSS used as an HTL [18,19]. Also, impressive efficiency of 13.82% is reported for FASnI_3_-based PSC with vertical Sn^+2^ gradient using PEDOT: PSS (HTL) and C_60_(ETL) [20].

The performance of the PSC is greatly influenced by the charge transport layers (CTL) in the structure. ETL (Electron Transport Layer) enhances the extraction of photogenerated electrons in the absorber while HTL (Hole Transport Layer) is responsible for the extraction of holes.

Spiro-OMeTAD and PEDOT: PSS as HTLs show high performance with FASnI_3_ but because of their organic nature they suffer from thermal stability and limited intrinsic hole transport. Both materials have a high cost, complex purification and multistep processing [21,22]. Different inorganic Cu-based materials (CuI, Cu_2_O, CuAlO_2_, CuSCN, etc.) are considered promising contenders for HTL because of their desirable characteristics like ease of manufacturing, low production cost, high charge mobility and chemical stability [23]. Similarly, Fullerene (Carbon Allotrope) derivatives such as C_60_ and Phenyl-C61-butyric acid methyl ester (PCBM) are widely used as ETL because of their thermal stability, and electron extraction properties [24,25]. For carbon-based materials like C_60_ and PCBM inverted p-i-n structure is preferred because of the smaller band gap of ETL and their low hysteresis behaviour in an inverted structure [26,27].

In this research, eight unique FASnI_3_-based solar cells are numerically modelled by using carbon-based ETLs (PCBM and C_60_) and Cu-based HTLs such as Cu_2_O (cuprous-Oxide), CuAlO_2_ (Copper Aluminum Oxide), CuSCN (Copper thiocyanate) and CuSbS_2_ (Copper Antimony Sulfide). A detailed study is carried out by analysing the effect of thickness, doping concentration, defect density, temperature, back contact electrodes, ARC coating and interface defects on the performance of solar cells. The optimized design parameters for each structure are identified and presented in this study. Moreover, the behaviour of different HTLs is closely analysed by comparing QE, Band Gap and Recombination graphs.

## 2. Methodology

### 2.1. Simulation tool

Extensive numerical modelling of the PSC structures is carried out through the simulation tool of SCAPS-1D. It has been developed by the Department of Electronics and Information systems (ELIS), University of Gent, Belgium. It is widely used for the simulation of different types of solar cells including perovskite, CdTe-based and silicon-based solar cells. The software is designed to model a maximum of seven layers for a single-cell structure. It assists in the modification of different solar cell parameters like band gap, defect density, doping, thickness, etc., to achieve better results. The numerical simulation in SCAPS is carried out by solving basic physical differential equations in a steady state such as the Poisson equation (equation (2.1)) and continuity equations (equations (2.2) and (2.3))

2.1 $$\frac{\partial^{2}\varphi }{\partial x^{2}}=\frac{q}{\varepsilon }[n-p+N_{A}-N_{D}+n_{t}-p_{t}],$$

2.2 $$\frac{i}{J}\frac{\partial J_{p}}{\partial x}+R_{p}(x)+G_{p}(x)=0,$$

2.3 $$\frac{i}{J}\frac{\partial J_{p}}{\partial x}+R_{p}(x)+G_{p}(x)=0,$$

2.4 $$Jn\;=\;qn\;(x)\mu_{n}E\;(x)+\;qD_{n}\;\;\frac{dn}{dx},$$

2.5 $$Jp\;=\;qp\;(x)\mu_{p}E\;(x)+\;qD_{p}\;\;\frac{dp}{dx},$$

2.6 $$L_{n}=\sqrt{D_{n\;}.\tau_{n}},$$

2.7 $$L_{p}=\sqrt{D_{p}.\tau_{p}},$$

2.8 $$\tau_{n}=\frac{1}{\sigma \;\times \;Nt\;\times \;v_{th\;}}$$

2.9 $$\mathrm{and}\;\tau_{p}=\frac{1}{\sigma \;\times \;Nt\;\times \;v_{th\;}}.$$

The symbols used in equations (2.1)–(2.8) are mentioned in table 1 along with units and descriptions.

**Table 1.** Description of the physical quantities used in the SCAPs simulator.

symbol	unit	description
φ	V	electro static potential
*N_A_*	cm^−3^	absorber defect density
*N_D_*	cm^−3^	donor defect density
*q*	C	electron charge
ε	F.cm^−1^	electric permittivity of material
*μ*	cm^2^/Vs	mobility of charge carrier
*p*	cm^−3^	hole density
*n*	cm^−3^	electron density
*G*_n_, *G*_p_	cm^3^s^−1^	generation rate of electron, generation rate of holes
*R*_n_, *R*_p_	cm^3^s^−1^	recombination rate of electrons, recombination rate of holes
*Dn*, *Dp*	cm^2^.s	diffusion coefficient
*Jn*, *Jp*	A.cm^−1^	electron current density, hole current density
*L*_n_, *L*_p_	μ.m	diffusion length of electron, diffusion length of hole
*τ*_n_. *τ*_p_	ns	carrier lifetime electron, carrier lifetime hole
*σ*	cm^−2^	electron capture cross-section, hole capture cross-section
*Nt*	cm^−3^	total defect density/bulk defect density
*v*_th_	V	thermal voltage

### 2.2. Device structure

**Figure 2.**
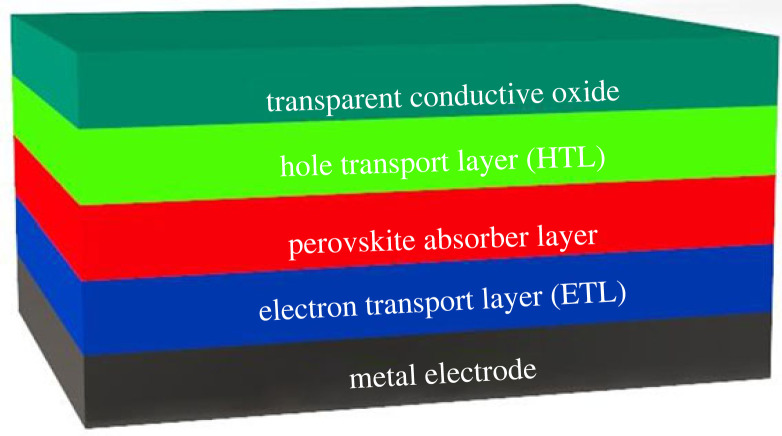
The basic inverted p-i-n solar cell device structure.

In this study, the inverted p-i-n structures of PSC are modelled as shown in figure 2. It consists of five main layers which are Anode/HTL/absorber/ETL/cathode. Transparent Conduction Oxide (TCO) is the top transparent layer which acts as an anode while the metal electrode is the back layer acting as a cathode. FASnI_3_ is an absorber layer and is sandwiched between p-doped HTL and n-doped ETL. The function of ETL and HTL is to enhance the charge extraction efficiency and facilitate charge transport (electrons and holes, respectively). In inverted p-i-n structures, ITO is preferred as TCO because of its lower electrical resistivity and higher transparency to the visible spectrum [28]. When light is absorbed by the perovskite layer photogenerated charge carriers are produced in an absorber. Electrons are transferred from the conduction band of the absorber to the conduction band of ETL; similarly, the holes are transferred from the valence band of the absorber to the valance band of the HTL. Metal-electrode (cathode) collects electrons from ETL while TCO (anode) collects holes from HTL. The eight novel structures are modelled in this study and presented in table 2.

**Table 2.** Group classification of cells.

Group 1 cells	Group 2 cells
ITO/Cu_2_O/FASnI_3_/PCBM/Al	ITO/Cu_2_O/FASnI_3_/C_60_/Al
ITO/CuAlO_2_/FASnI_3_/PCBM/Al	ITO/CuAlO_2_/FASnI_3_/C_60_/Al
ITO/CuSCN/FASnI_3_/PCBM/Al	ITO/CuSCN/FASnI_3_/C_60_/Al
ITO/CuSbS2/FASnI_3_/PCBM/Al	ITO/CuSbS2/FASnI_3_/C_60_/Al

### 2.3. Device modelling

The design parameters for all the layers (ETL, HTL and FASnI_3_) have been collected from multiple experimental plus theoretical studies and mentioned in table 3 [10,29–31]. Al with the work function of 4.2 is used as back contact metal while ITO with a work function of 5 is used as front contact in this study. The initial thickness for all CTL is kept at 150 nm. The defect density of ETLs and HTLs is kept at 10^14^ cm^−3^. For FASnI_3_ defect density of 2 × 10^15^ is considered to obtain the carrier lifetime of 2.5 ns which in the literature ranges from 1 to 4 ns [16]. As for the interface defects, single energetic distribution is incorporated alongside other parameters mentioned in table 4. All the simulations are carried out under AM 1.5 radiation with solar irradiance of 1000 W m^–2^ and at room temperature (300 K).

**Table 3.** Design parameters for all active layers.

parameters	FASnI_3_	PCBM	C_60_	Cu_2_O	CuAlO_2_	CuSCN	CuSbS_2_
thickness (nm)	300	150	150	150	150	150	150
bandgap *E*_g_ (eV)	1.41	2	1.7	2.17	3.46	3.4	1.58
electron affinity *χ* (eV)	3.9	3.9	3.9	3.2	2.5	2.1	4.2
dielectric permittivity	8.2	4	4.2	7.1	60	10	14.6
CB effective density of states (cm^−3^)	10^18^	10^21^	8 × 10^19^	2.5 × 10^20^	2 × 10^20^	2.5 × 10^18^	2 × 10^18^
VB effective density of state *N_v_* (cm^−3^)	10^18^	2 × 10^20^	8 × 10^19^	2.5 × 10^20^	1 × 10^22^	1.8 × 10^19^	1 × 10^18^
electron mobility *μ*_n_ (cm^2^ Vs^−1^)	22	10^−2^	8 × 10^−2^	200	2	2 × 10^−4^	49
hole mobility *μ*_p_ (cm^2^ Vs^−1^)	22	10^−2^	3.5 × 10^−3^	8.6 × 10^3^	8.6	2 × 10^−4^	49
donor density *N_D_* (cm^−3^)	0	10^20^	2.6 × 10^17^	0	0	0	0
acceptor density *N_A_* (cm^−3^)	7 × 10^16^	0	0	10^19^	10^20^	10^17^	10^18^

**Table 4.** Absorber defect density and interface defects parameters.

	ETL/absorber	HTL/absorber	FASnI_3_
defect type	neutral	neutral	neutral
capture cross-section of electrons and holes (cm^−2^)	1 × 10^−15^	1 × 10^−15^	1 × 10^−15^
energetic distribution	single	single	gaussian
energy level w.r.t to *E_v_*	0.6	0.6	0.6
characteristic energy (eV)	—	—	0.1
total density (cm^−3^)	1 × 10^10^	1 × 10^10^	2 × 10^15^

**Figure 3.**
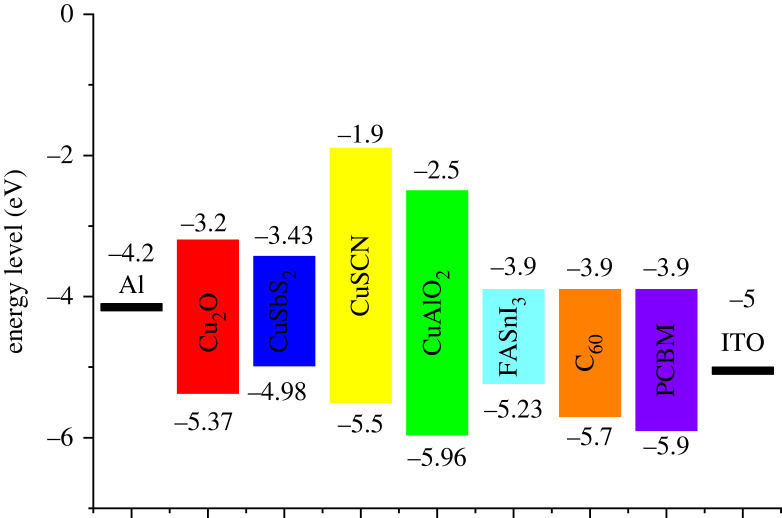
Energy level diagram for different active layers of PSC.

**Figure 4.**
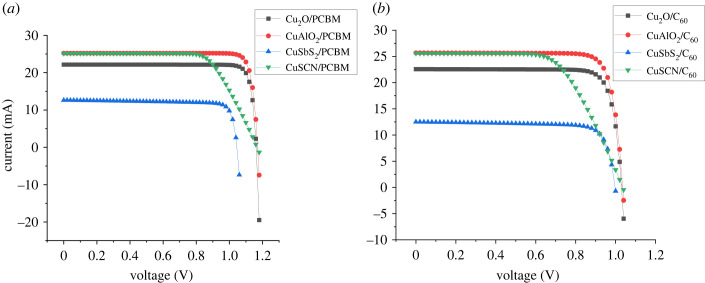
I–V curves for simulated cells in (*a*) Group 1 and (*b*) Group 2 cells.

The energy level diagram for all the layers is shown in figure 3. The IV cure obtained for the different structures is shown in figure 4 while the characteristics are presented in table 5.

**Table 5.** Initial Performance Parameters for non-optimized cells.

cells	*V*_oc_	*J*_sc_	FF	PCE %
ITO/Cu_2_O/FASnI_3_/PCBM/Al	1.127	22.129	86.634	21.608
ITO/CuAlO_2_/FASnI_3_/PCBM/Al	1.133	25.200	86.197	24.611
ITO/CuSbS_2_/FASnI_3_/PCBM/Al	1.043	12.618	82.322	10.839
ITO/CuSCN/FASnI_3_/PCBM/Al	1.167	25.178	69.966	20.569
ITO/Cu_2_O/FASnI_3_/C_60_/Al	1.029	22.524	83.410	19.330
ITO/CuAlO_2_/FASnI_3_/C_60_/Al	1.034	25.633	82.892	21.979
ITO/CuSbS_2_/FASnI_3_/C_60_/Al	0.996	12.491	79.475	9.897
ITO/CuSCN/FASnI_3_/C_60_/Al	1.035	25.610	62.878	16.675

### 2.4. Device behaviour

#### 2.4.1. IV characteristics

The squareness of the I–V curve or in other words FF determines the parasitic losses (series and shunt losses) present in solar cells. The higher the FF values the lower the parasitic losses. FF is strongly related to series resistance [32]. An increase in series resistance results in a decrease in FF.

2.10 $$\mathrm{FF}={\mathrm{FF}}_{o}\left(1-R_{s}\frac{J_{\mathrm{sc}}}{V_{\mathrm{oc}}}\right),$$

where *R_s_* is series resistance, FF*_o_* is the value of *FF* at *R_s_* = 0 Ω cm^2^. *R_s_* includes TCO series resistance, resistance in charge transport at the interface, and the bulk of the solar cell.

Among the proposed cells for given ETL (C_60_ or PCBM), Cu_2_O-based cells show better IV behaviour in figure 4 followed by CuAlO_2_, while CuSCN shows clear signs of series resistance.

#### 2.4.2. Optical absorption

In p-i-n structures, HTL acts as a front layer after TCO. For maximum photons to reach the perovskite layer, it is required that HTL possesses a minimum optical absorption with maximum transmittance. Materials with low absorption coefficients possess high transmittance values. The absorbance and transmittance of a material can be related by the following equation:

2.11 $$\alpha =\frac{\;\ln(1/T)}{d}.$$

Where *α* is an absorption coefficient, *T* represents transmittivity and *d* is the film thickness.

The absorption coefficient depends upon the optical band gap of the material. Materials with higher optical band gap values have low optical absorption. The absorption coefficient of material as a function of wavelength can be related to the optical band gap of material by the following equation:

2.12 $$\alpha (\lambda )=\left(A+\frac{B}{h\nu }\right)\surd (h\nu -E_{g}),$$

where *hν* represents the photon energy and *E_g_* represents the band gap of a material.

**Figure 5.**
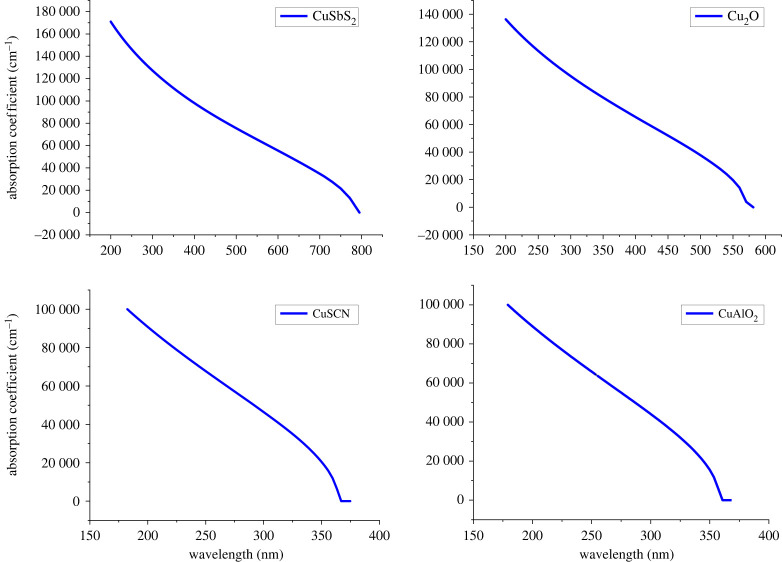
The optical absorption of the HTLs.

Materials with large band gaps have transmissivity for low-energy photons, allowing them to pass through without being absorbed. Absorption in films in transparent regions may occur due to scattering of light, defect absorption, and multi-phonon absorption or Urbach tail [33–35]. Figure 5 shows that CuSCN and CuAlO_2_ have high transmittance to the photons with a wavelength greater than 350 nm which include the visible spectrum (400–700 nm). Among the above-mentioned HTLs CuAlO_2_ (*E_g_* = 3.46) has maximum transmittance followed by CuSCN (*E_g_* = 3.4), Cu_2_O (*E_g_* = 2.17) and CuSbS_2_ (*E_g_* = 1.58), respectively. The transmittance of a material depends on a variety of factors including preparation methods, deposition methods and also thickness of the layer [33,36].

#### 2.4.3. Quantum efficiency

QE or IPCE (incident photon to current efficiency) is the measure of electrons collected by a solar cell in response to the incident photons at a certain wavelength. QE at different wavelengths represents the spectral response of different solar cells to the incident light. It can be calculated from the following equation:

2.13 $$\mathrm{QE}=\frac{\mathrm{no}.\;\;\mathrm{of}\;\mathrm{reacted}\;\mathrm{electrons}}{\mathrm{no}.\;\mathrm{of}\;\mathrm{incident}\;\mathrm{photons}}.$$

**Figure 6.**
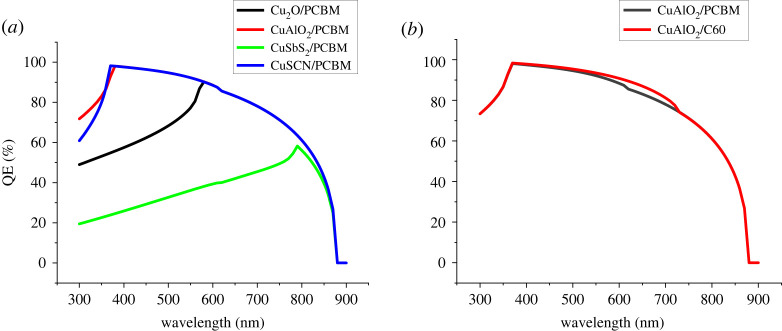
Effect of different CTL on QE of the cell (*a*) HTL and (*b*) ETL.

Figure 6*a,b* shows different QE peaks for the solar cells on the basis of HTLs and ETL listed in table 4. The shift in peaks can be closely related to the absorption curves of HTLs in figure 5. The QE of the charge transport layer in figure 6 rises for the wavelengths where the absorption coefficient of the material is minimum. The smaller the absorption coefficient the higher the transparency and thus more photons reach the absorber layer through HTLs which increases QE.

#### 2.4.4. Energy band alignment

**Figure 7.**
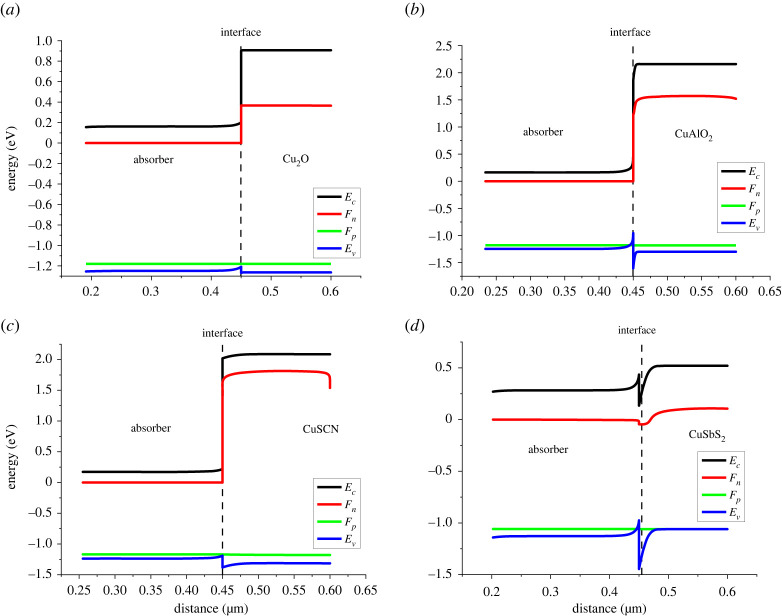
Band alignment at (*a*) absorber/Cu_2_O, (*b*) absorber/CuAlO_2_, (*c*) absorber/CuSCN interfaces and (*d*) absorber/CuSbS_2_.

Band gap alignment plays an important role in determining the performance of solar cells. It determines how well an absorber performs with a set of CTL. As can be seen in figure 3 each material has different energy levels, therefore forming different energy band alignments and band offsets. Figure 7 shows the band gap alignment at the absorber/HTL interface. The function of HTL is not only to transport holes from the absorber but also to block electrons from the absorber [37]. For the efficient transport of holes, minimum valence band offset (VBO) is required at the absorber/HTL interface, while for blocking electrons from the absorber, maximum conduction band offset (CBO) is required.

The key energy levels (figure 7) in a photovoltaic cell are the conduction band (*E_c_*), the Fermi level for the n-type material (*F*_n_), the Fermi level for the p-type material (*F*_p_) and the valence band (*E_v_*).

— The conduction band (*E_c_*) is highest energy level in the solid where electrons can freely move and is made of LUMO (lowest molecular orbital) of semiconductor.
— The Fermi level for the n-type material (*F*_n_) is the energy level at which the probability of occupation of an electron is 0.5. The significance of this level is that it separates the energy levels where electrons are likely to be found from the energy levels where they are unlikely to be found. In an n-type semiconductor or ETLs, the Fermi level is near the conduction band due to the excess of electrons.
— Similarly at Fermi level for p-type semiconductors (*F*_p_) the probability of finding holes occupation is 0.5 at absolute temperature. In a p-type semiconductor or HTLs, the Fermi level is close to the valence band because of excess holes.
— The valence band (*E_v_*) is the lowest energy level in the solid where electrons are tightly bound to the atoms and cannot move freely. In a solar cell, the valence band is typically made up of the highest occupied molecular orbital (HOMO) of the semiconductor material.

These energy bands are arranged in such a way as to facilitate the charge transport in solar cells. When light falls on perovskite (absorber layer) e-h pairs are produced as the electrons are excited from valence to conduction band. The electrons in the conduction band of absorber layer move conduction n-type material (ETL); similarly the holes in the absorber layer move to the valence band of absorber to the valence band of p-type material. These charge carriers are then collected by electrodes.

The Fermi levels of the n-type and p-type layers provide a built-in potential barrier at heterojunction. This potential barrier ensures that the electrons and holes are separated and prevented from recombining. The built-in potential barrier also creates a driving force for the electrons and holes to move towards the electrodes and contribute to the overall current generated by the solar cell.

Two types of discontinuities occur in response to band offset at the interface: positive and negative. If the absorber's conduction band is higher than the conduction band of the CTL, it forms a negative cliff (–CBO) at the heterojunction. While, if the absorber's conduction band is beneath the conduction band of the CTL, it forms a positive spike (+CBO) at the heterojunction [38–40]. Similarly, cliff and spike are also formed by the valance band at the heterojunction. VBO and CBO at the absorber/HTL interface can be calculated through the following equations:

2.14 $$\mathrm{VBO}=\chi_{\mathrm{HTL}}-\chi_{\mathrm{Per}}+E_{g_{\mathrm{HTL}}\;}-E_{g_{\mathrm{Perov}}\;}$$

and

2.15 $$\mathrm{CBO}=\chi_{\mathrm{Per}}-\;\chi_{\mathrm{HTL}}.$$

For all the HTLs spike discontinuity was observed at the valence band shown in table 6 and figure 7. Cu_2_O forms the best band alignment with a smaller VBO (spike) of 0.06 eV thus facilitating the hole transport and higher CBO value of 0.7 eV, blocking the leaked electrons from the absorber. This ensures the smooth flow of holes and the blockage of electrons. The small spike, i.e. with VBO < 0.4 eV, facilitates the flow of holes at the interface because of high built-in potential (*V*_bi_) values, thus resulting in a drop in recombination [38,41]. The large spike (VBO > 0.4 eV) hinders the flow of the holes because it forms low *V*_bi_ values at the interface and resulting in low *V*_oc_ values [42]. *V*_bi_ can be related to the *V*_oc_ by the following relation:

2.16 $$V_{\mathrm{oc}}=V_{\mathrm{bi}}-\frac{nKT}{q}\ln\frac{J_{o}}{J_{L}}.$$

**Table 6.** VBO and CBO at the absorber/HTL interface.

absorber/HTL interface	CBO	VBO
FASnI_3_/Cu_2_O	+0.7	+0.06
FASnI_3_/CuAlO_2_	+1.4	+0.65
FASnI_3_/CuSCN	+1.8	+0.19
FASnI_3_/CuSbS_2_	−0.3	+0.47

As can be seen in table 4 large spikes (VBO > 0.4 eV) are observed at the absorber/CuSbS_2_ and absorber/CuAlO_2_ interface. The worst alignment can be observed at the absorber/CuSbS_2_ interface because of large spike of +0.47 eV, thus hindering the flow of holes and smaller CBO of (−0.3 eV) which might result in the leakage of electrons towards the HTL side.

**Figure 8.**
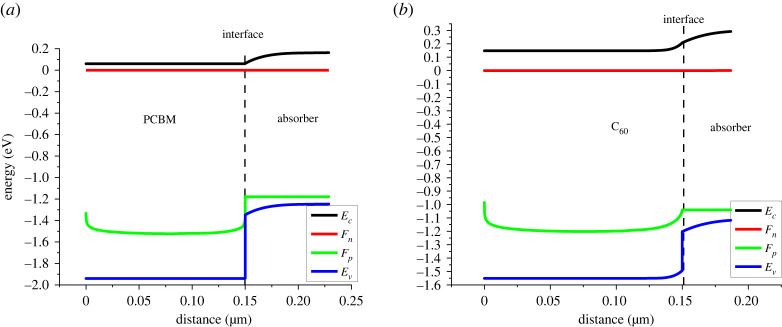
Band alignment at (*a*) PCBM/absorber interface and (*b*) C_60_/absorber interface.

The main purpose of ETL is to facilitate the smooth transfer of electrons as well as the blockage of holes from the absorber layer which can be controlled by band alignment. Figure 8 shows band gap alignment at ETL and absorber interface. For smooth electron transport to take place minimum CBO at the ETL/absorber interface is required and to block leaked holes from the absorber maximum VBO is preferred at the interface. The VBO and CBO at the ETL/absorber interface are mentioned in table 7.

**Table 7.** VBO and CBO at ETL/absorber interface.

ETL/absorber interface	CBO	VBO
PCBM/FASnI_3_	0	+0.59
C_60_/FASnI_3_	0	+0.29

#### 2.4.5. Electric potential at heterojunction

**Figure 9.**
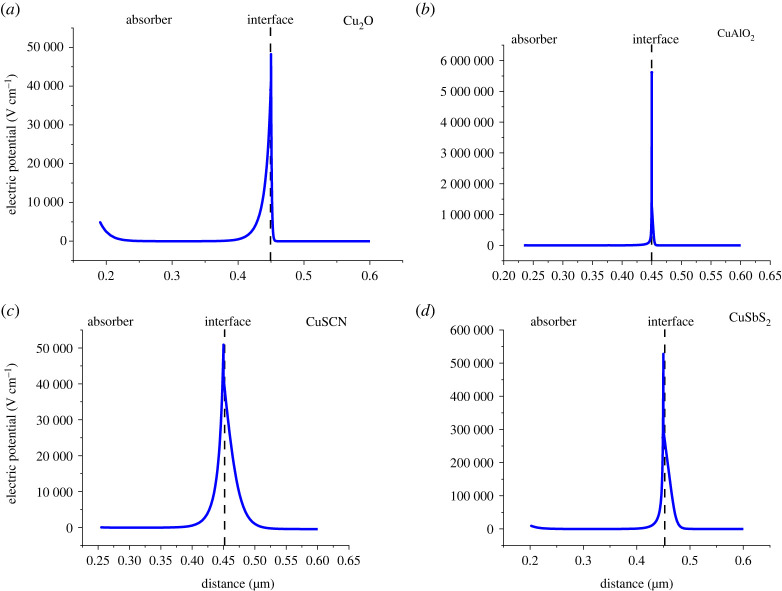
Electric potential at (*a*) absorber/Cu_2_O, (*b*) absorber/CuAlO_2_, (*c*) absorber/CuSCN and (*d*) absorber/CuSbS_2_ interfaces.

**Figure 10.**
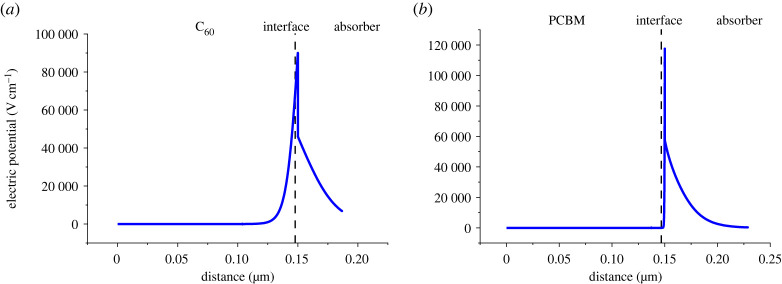
Electric potential at (*a*) C_60_/absorber interface and (*b*) PCBM/absorber interface.

Electron and hole transport layers play an important role in facilitating the transport of charge carriers produced in the absorber layer. The charge carriers produced at the absorber layer are transferred to the ETL or HTL as a result of a strong electric potential/electric field at the absorber/CTL interface due to band alignment. Figures 9 and 10 show the electric potential values at the absorber/HTL and ETL/absorber interface, respectively. The high values of the electric field increase the conductivity of charge carriers and reduce recombination leading to a high value of *V*_oc_. The electric field at the interface is caused by quasi-fermi level splitting at the absorber/CTL interface [43]. When the difference between fermi levels increases the electric field at the interface increases leading to high *V*_oc_ [44]. Fermi level difference/splitting ($\Delta E_{F})$ is related to *V*_oc_ through the following equation:

2.17 $$\Delta E_{F}=qV_{oc}.$$

As the results show in figure 9*b,* the absorber/CuAlO_2_ shows the highest electric potential values at the interface.

#### 2.4.6. Recombination at heterojunction

**Figure 11.**
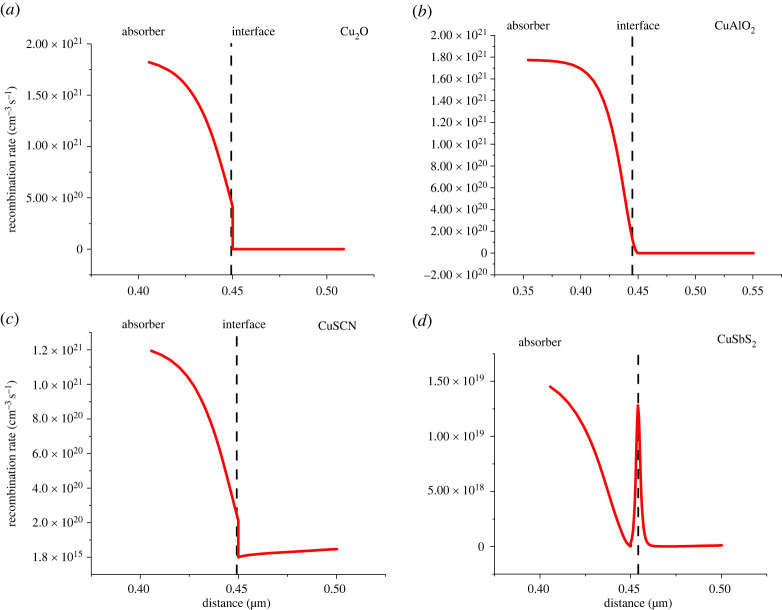
Recombination at (*a*) absorber/Cu_2_O, (*b*) absorber/CuAlO_2_, (*c*) absorber/CuSCN interfaces and (*d*) absorber/CuSbS_2_.

Recombination at the interfaces is highly dependent upon the VBO and CBO values. As values for VBO mentioned in table 6 large spikes occur at the absorber/CuSbS_2_ and absorber/CuAlO_2_ interface. Because of the large spike the charges accumulate at the interface resulting in recombination rise as can be seen in figure 11*d*. However, the recombination decreases at absorber/CuAlO_2_ as shown in figure 11*b*, despite having a larger spike, because CuAlO_2_ is highly doped (*N_A_* = 10^20^ cm^−3^). As a result of this Fermi level splitting at the interface is high which develops a strong electric field at the interface as shown in figure 9*b,* thus improving charge transport and reducing the recombination rate [44].

**Figure 12.**
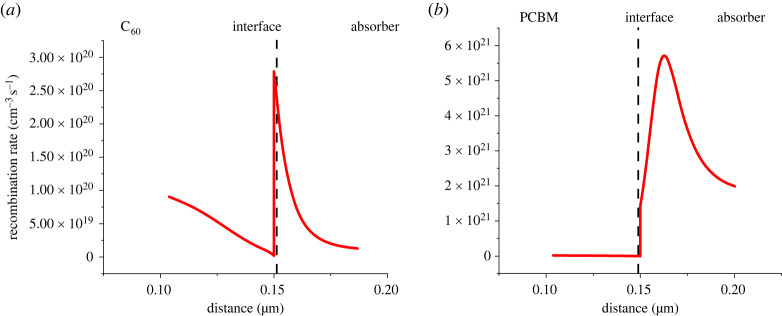
Recombination at (*a*) C_60_/absorber interface and (*b*) PCBM/absorber interface.

Figure 12 shows a rise in recombination rate at the C_60_/absorber interface while at the PCBM/absorber interface it is decreasing. The increase in recombination at the C_60_/absorber interface is because the VBO (0.29 eV) is less than 0.4 eV, because of which the holes are leaking towards the ETL which is increasing the recombination.

## 3. Results and discussion

### 3.1. Absorber and charge transport layers thickness optimization

**Figure 13.**
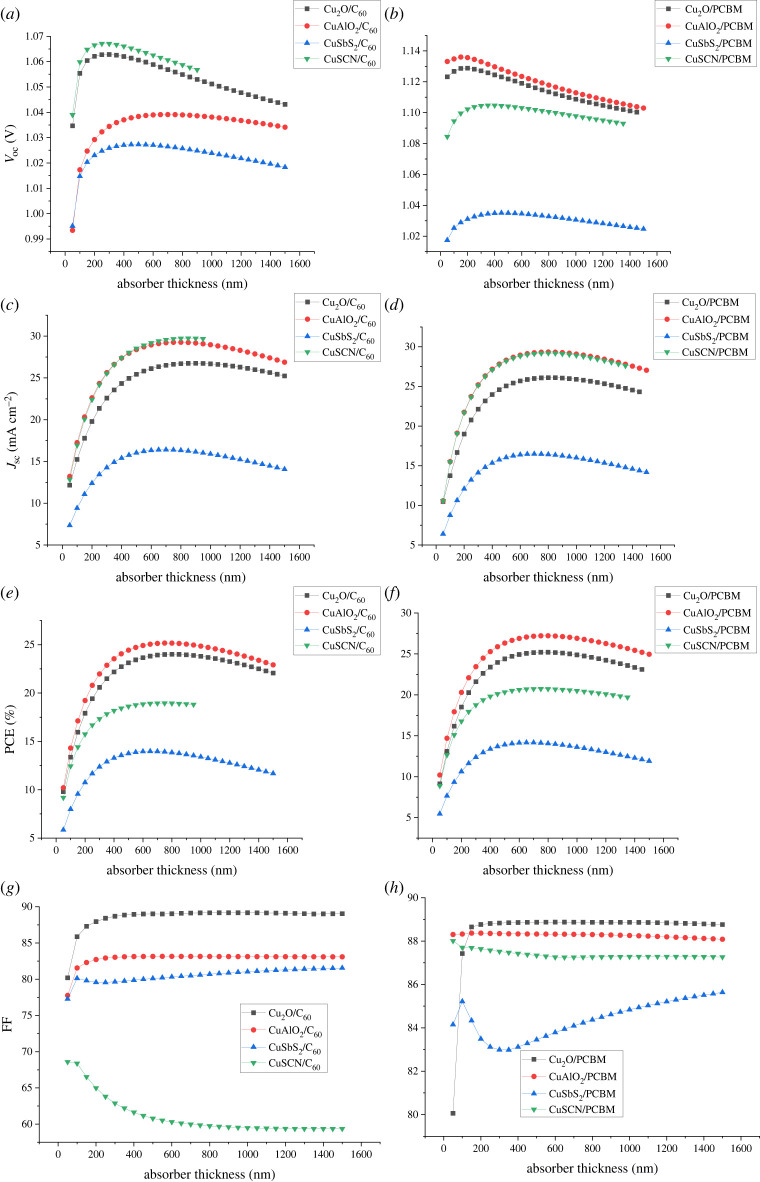
Effect of absorber thickness on (*a,b*) *V*_oc_, (*c,d*) *J*_sc_, (*e,f*) PCE and (*g,h*) FF.

Absorber layer thickness has a great effect on the performance of solar cells, influencing absorption, generation and charge transport. The effect of absorber thickness is analysed by varying thickness from 50 nm to 1500 nm (50 nm increment) while keeping all the other device parameters shown in table 3 constant. The optimum results were observed at different thickness values mentioned in table 8. With the absorber thickness increase, the increase in *V*_oc_, PCE, FF and *J*_sc_ is observed as shown in figure 13. A thicker absorber layer can absorb a wide range of photons resulting in more charge carrier generation which leads to the increase in *J*_sc_. It also causes a decrease in the effective band gap, thus photons of larger wavelengths are also absorbed [45,46]. When the thickness is low, the photons of larger wavelengths and low energy are not absorbed. Therefore, a moderate thickness of the absorber is required. The absorber thickness depends greatly upon the diffusion length and absorption coefficient of the material.

After achieving optimum thickness, further increase leads to saturation and eventually a decrease in PCE and FF. This is due to the absorber thickness exceeding the carrier diffusion length, making the charge carriers unable to reach the CTL resulting in a decrease in performance because of an increase in recombination. The thickness of the absorber can be increased up to the diffusion length of a material [16]. The optimum results were observed at different thickness values mentioned in table 8.

The CTL (ETL and HTL) in solar cells not only serve the purpose of charge transport from the absorber to the respective electrodes but also play the role of separation layer between the absorber and electrode [47].

**Figure 14.**
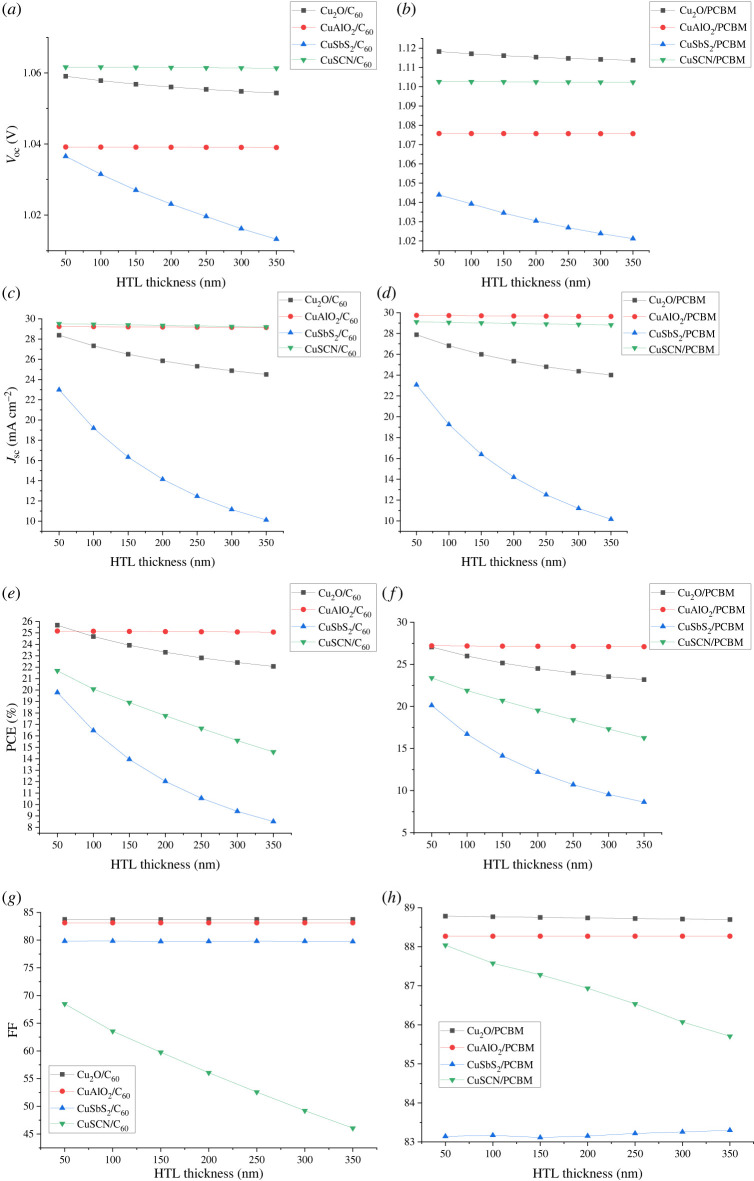
Effect of HTL thickness on (*a,b*) *V*_oc_, (*c,d*) *J*_sc_, (*e,f*) PCE and (*g,h*) FF.

**Figure 15.**
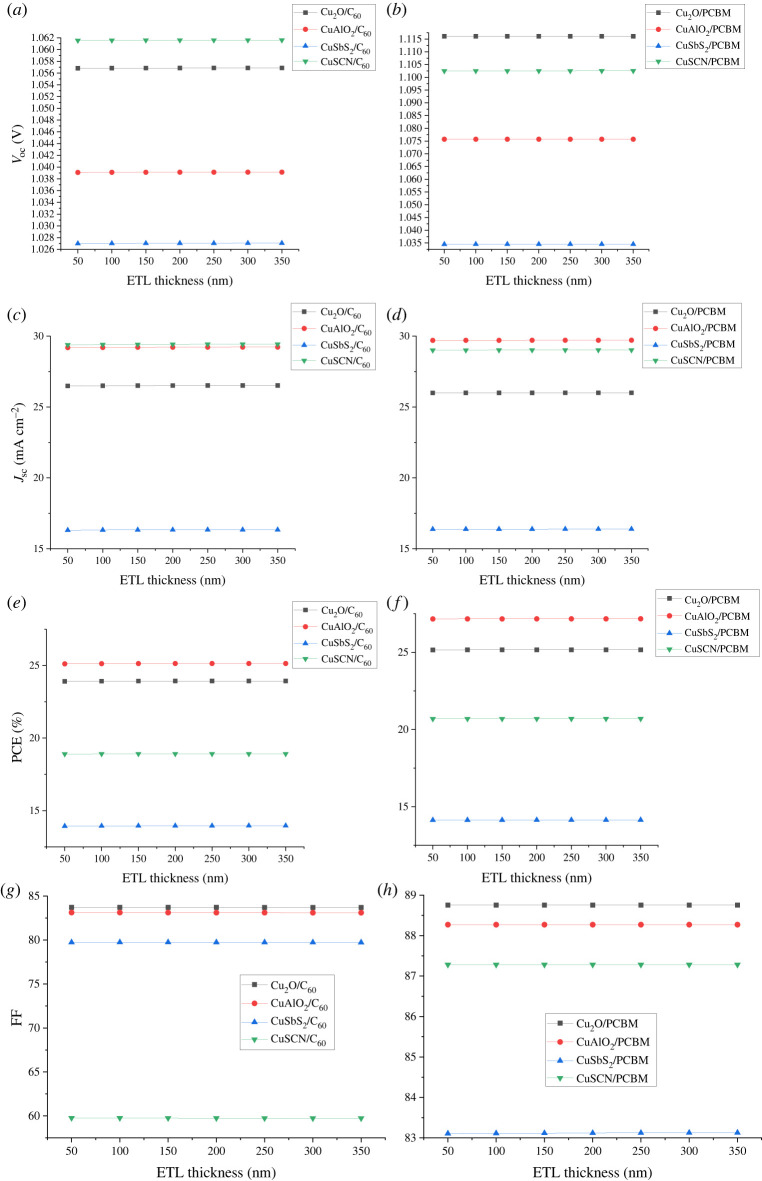
Effect of ETL thickness on (*a,b*) *V*_oc_, (*c,d*) *J*_sc_, (*e,f*) PCE and (*g,h*) FF.

After the optimization of absorber thickness, ETL thickness was optimized by varying thickness from 50 nm to 350 nm keeping the absorber and HTL thickness constant. Once the optimum thickness for ETL was determined, HTL thickness was optimized keeping both ETL and absorber at optimized thickness values. In p-i-n inverted solar cells, HTL being the front layer has a major effect on performance which decreases with an increase in thickness. The decrease in performance is because of the decrease in transparency with thickness. While ETL thickness has a negligible effect on solar cell performance as shown in figures 14 and 15. So, for all calculations, ETL thickness is kept at 100 nm to achieve the purpose of separating the layer. By increasing the thickness of HTL, the series resistance between the absorber and HTL increases, resulting in a decrease in *V*_oc_ and leading to a drop in FF, *J*_sc_ and PCE.

### 3.2. Optimization of absorber and charge transport layers doping

**Figure 16.**
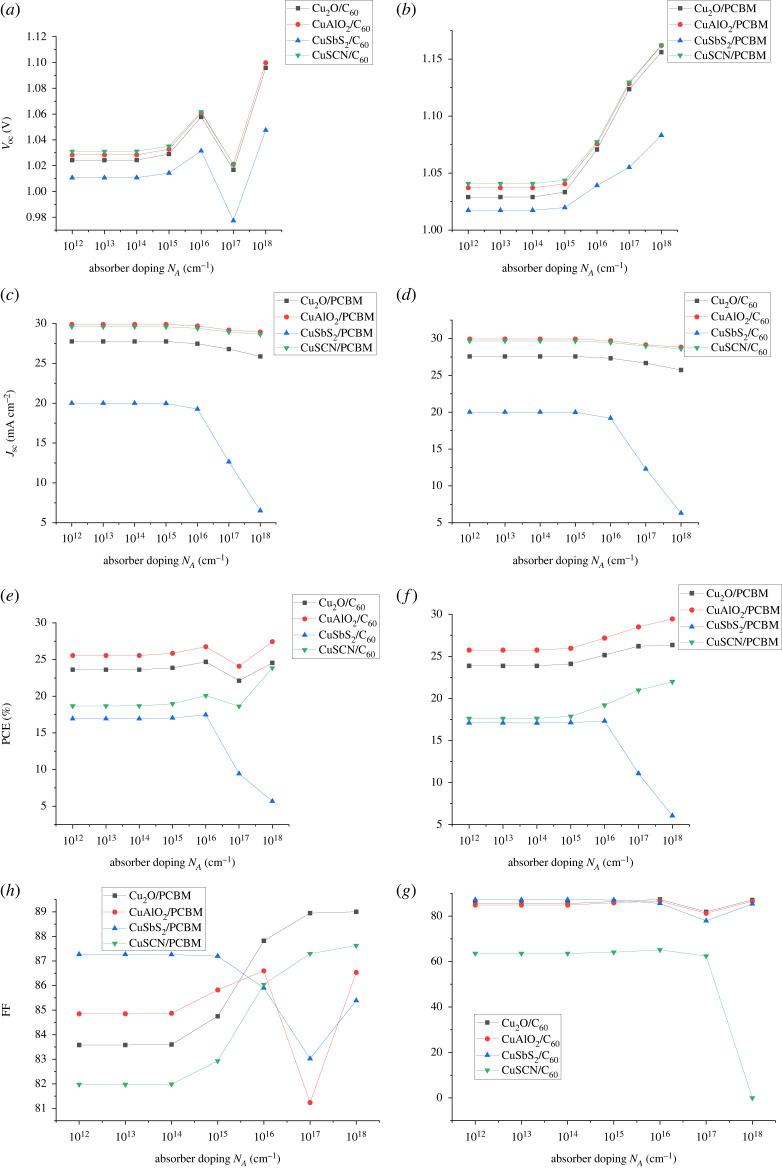
Effect of absorber doping on (*a,b*) *V*_oc_, (*c,d*) *J*_sc_, (*e,f*) PCE and (*g,h*) FF.

To investigate the optimization of absorber doping, its acceptor doping density N_A_ is varied from 10^12^ to 10^18^ cm^−1^ while keeping the thickness of an absorber and CTLs at optimized values. Absorber doping increases the performance of solar cells because it increases the density of majority charge carriers and hence improves conductivity and reducing recombination rate [48,49]. Figure 16 shows that when the doping is increased from 10^14^ cm^−1^ to 10^16^ cm^−1^, the performance of cells is increasing because of an increase in the electric field across the absorber interface. The electric field facilitates the charge separation which translates to an increase in PCE and *V*_oc_ [47]. However, as *N_A_* is increased above 10^16^ for some cells, a sudden drop is observed which is implied by the fact that at higher doping values auger recombination dominates. Increasing *N_A_* leads to suppression in charge transportation and a decrease in charge mobilities (*μ*_n_ and *μ*_p_) due to an increase in ionized impurity scattering [50]. The sudden increase in *V*_oc_ PCE and *J*_sc_ in a majority of cells at very high doping (above 10^17^ cm^−1^) absorber starts losing its semiconductor behaviour and adopting metallic properties [47]. The optimum doping of absorber of 10^16^ was selected for all the cells.

**Figure 17.**
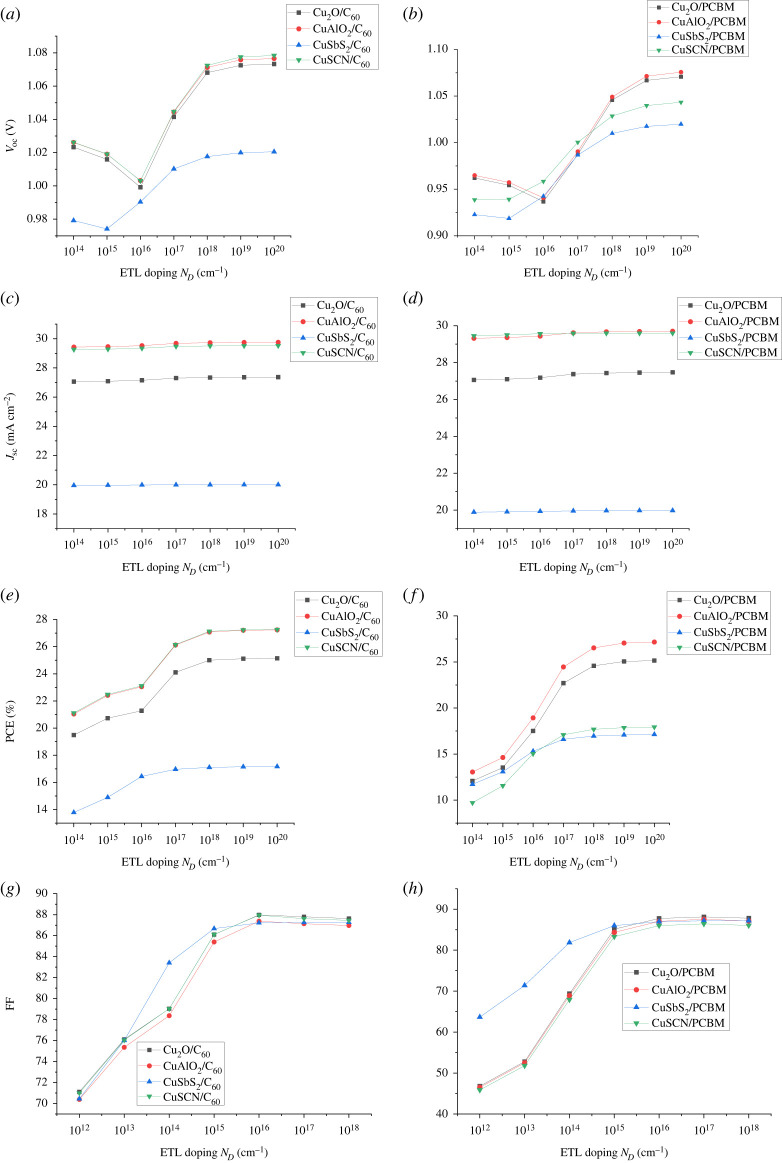
Effect of ETL doping (N_D_) on (*a,b*) *V*_oc_, (*c,d*) *J*_sc_, (*e,f*) PCE and (*g,h*) FF.

**Figure 18.**
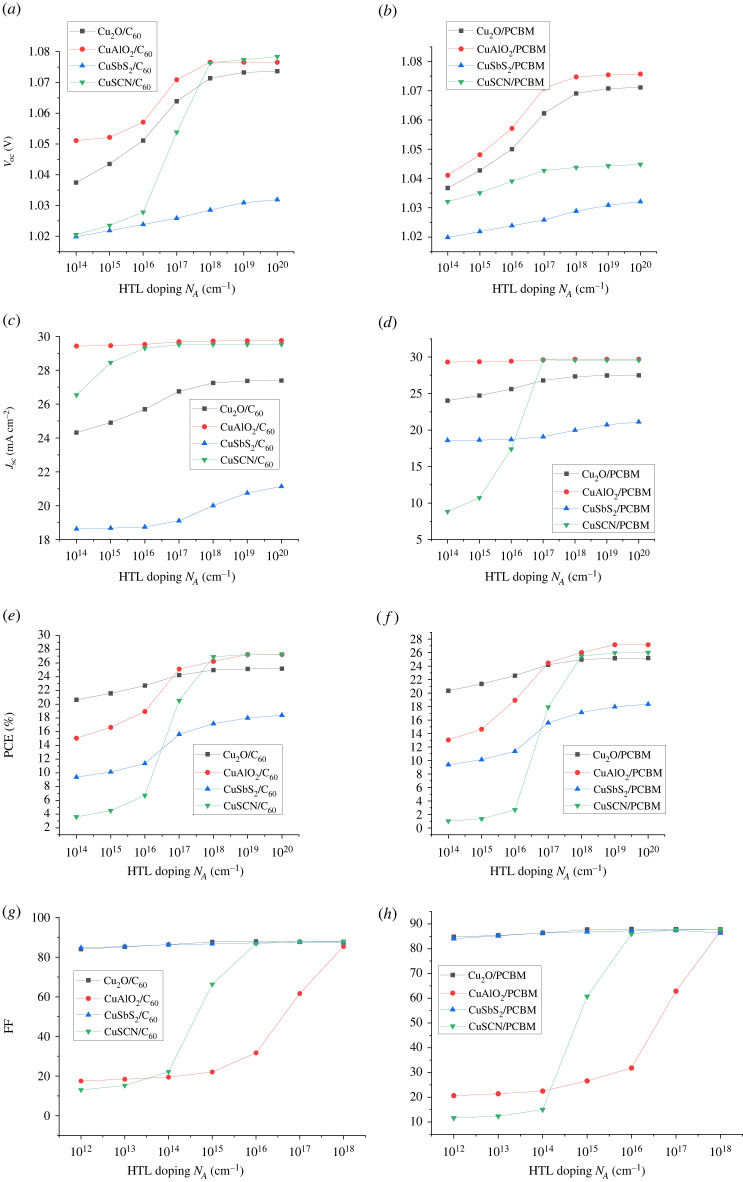
Effect of HTL doping (N_D_) on (*a,b*) *V*_oc_, (*c,d*) *J*_sc_, (*e,f*) PCE and (*g,h*) FF.

After the optimization of absorber doping the optimization of ETL and HTL is performed. The doping density of ETL (N_D_) and HTL (N_A_) is varied from 10^14^ cm^−1^ to 10^20^ cm^−1^. Doping of CTL improves the conductivity of charge by enhancing the electric field at the absorber and CTL interface. The increase in doping increases the fermi level difference at the absorber/CTL interface developing a strong electric field that facilitates the charge transport to respective electrodes [51]. The electric field at the ETL/absorber interface helps in electron extraction, i.e. facilitating the transfer of electrons (majority charge carriers) and blocking holes (minority charge carriers). Similarly, the electric field at the absorber/HTL interface attracts holes (majority charge carriers) toward HTL while repelling the electrons (minority charge carriers). The increase in FF, PCE, *V*_oc_ and *J*_sc_ as a result of the increase in ETL and HTL doping can be seen in figures 17 and 18, respectively. The optimized values for CTLs (ETL and HTL) are taken as 10^20^ cm^−1^.

#### 3.2.1. Optimized solar cell parameters

The optimized results obtained as a consequence of the thickness and doping optimization are mentioned in table 8. As mentioned in the Results, the optimized thickness value for all ETLs is considered 100 nm. The absorber doping for all the cells was selected to be 10^16^ cm^−1^ while the CTLs doping is taken at 10^20^ cm^−1^.

**Table 8.** Performance parameters of optimized cells.

cells	absorber thickness (nm)	HTL thickness (nm)	*V*_oc_	*J*_sc_	FF	PCE
ITO/Cu_2_O/FASnI_3_/PCBM/Al	750	100	1.071	27.494	85.519	25.185
ITO/CuAlO_2_/FASnI_3_/PCBM/Al	700	150	1.076	29.702	85.019	27.164
ITO/CuSbS2/FASnI_3_/PCBM/Al	600	100	1.034	21.110	84.015	18.340
ITO/CuSCN/FASnI_3_/PCBM/Al	550	100	1.0438	29.572	84.217	25.996
ITO/Cu_2_O/FASnI_3_/C_60_/Al	700	100	1.074	27.389	85.587	25.169
ITO/CuAlO_2_/FASnI_3_/C_60_/Al	700	150	1.077	29.753	85.027	27.235
ITO/CuSbS_2_/FASnI_3_/C_60_/Al	600	100	1.035	21.135	84.028	18.386
ITO/CuSCN/FASnI_3_/C_60_/Al	650	100	1.078	29.524	85.621	27.260

### 3.3. Effect of absorber defect density

Defect density is an important parameter for determining the performance of an absorber. The important processes like recombination, generation and absorption that occur in the absorber layer are highly dependent on its defect density and film quality [29,52]. Poor film quality results in an increase in defect density. Defects act as recombination centres and enhance the recombination rate. In PSC, Trap-assisted Shockley–Read–Hall (SRH) recombination (non-radiative recombination) is dominant as compared to other types of recombination [53,54] and is represented by the following equation:

3.1 $$R_{\mathrm{SRH}}=\frac{np-n_{i}^{2}}{\tau (\;p+n\;2ni\cosh(E_{i\;}-E_{t\;}/KT))}.$$

**Figure 19.**
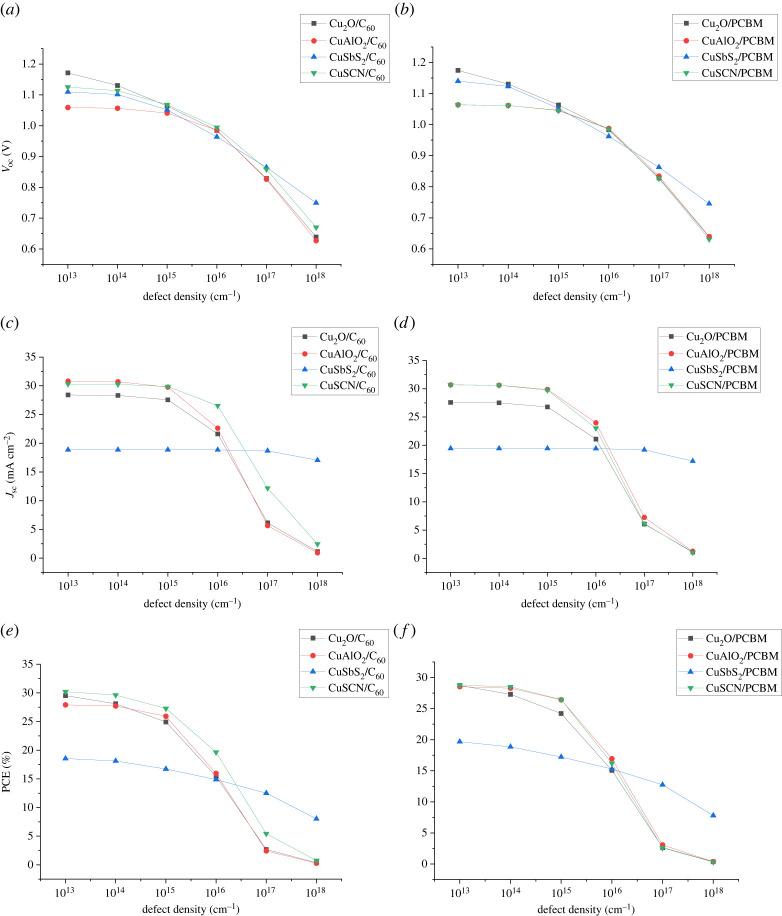
Effect of variation in absorber defect density on (*a,b*) *V*_oc_, (*c,d*) *J*_sc_ and (*e,f*) PCE.

This type of recombination occurs through localized energy states called traps between valance and conduction bands created as a result of doping or defect in the crystal lattice. An increase in defect density (*N_t_*) results in a decrease in a carrier lifetime (*τ*) of charge carriers which results in a decrease in carrier diffusion length and an increase in non-radiative recombination (*R*_SRH_) which results in the degradation of the PCE of the cell [55]. The effect of defect density on an absorber layer is analysed by varying its defect density from 10^13^ to 10^18^ (cm^−1^), keeping all optimized thickness and doping values constant as shown in figure 19.

### 3.4. Effect of interface defects

Interface defects arise in PSCs at the interfaces of the absorber and CTL because of the energy level alignment of both layers in addition to trap defect densities. Recombination through traps depends upon a variety of factors including trap density (*N_t_*) energy level of trap state (*E_t_*) and electron and hole cross-section [56]. Two types of defects occur because of the energy level of trap states. Shallow defects occur when *E_t_* lies close to *E_V_* or *E_c_* while defects occur when the defect lies close to mid of *E_g_* [57].

**Figure 20.**
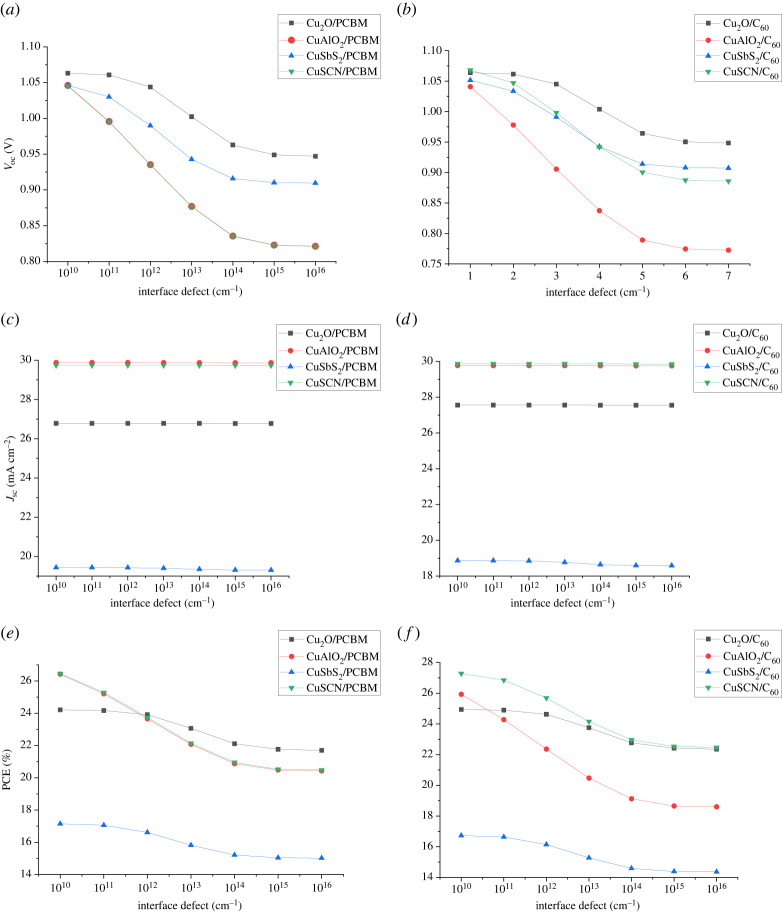
Effect of interface defect density at the ETL/absorber interface on (*a,b*) *V*_oc_, (*c,d*) *J*_sc_ and (*e,f*) PCE.

**Figure 21.**
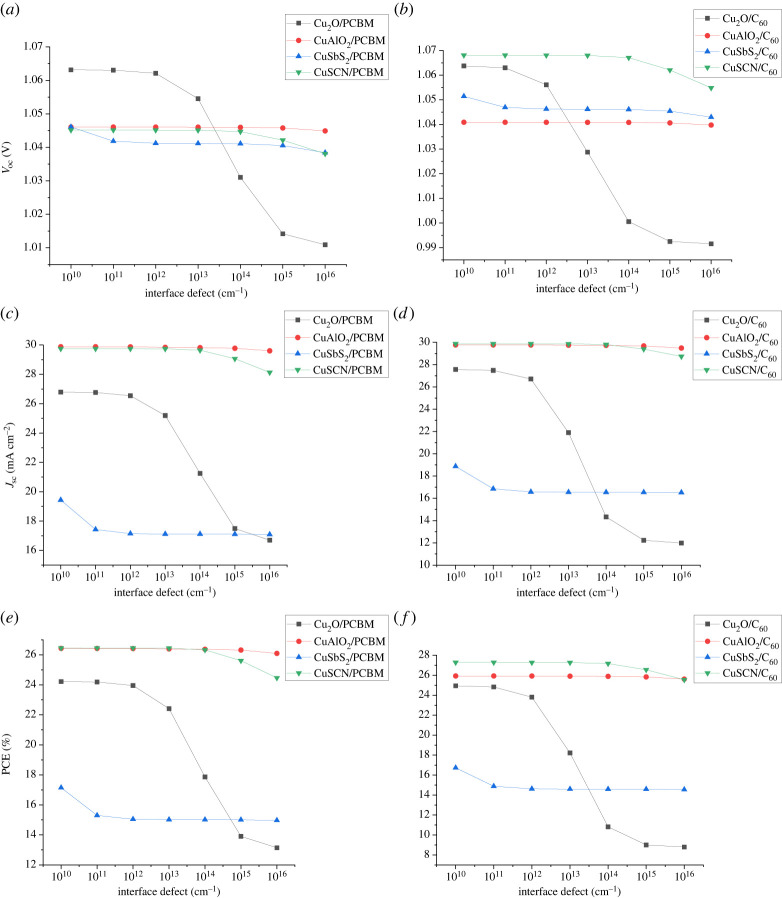
Effect of interface defect density at the absorber/HTL interface on (*a,b*) *V*_oc_, (*c,d*) *J*_sc_ and (*e,f*) PCE.

To observe the effect of interface defect density, the energy level of the trap is considered 0.6 eV above *E_v_*. The recombination in an interface is evaluated by the Pauwels–Vanhoutte theory which is an extension of SRH theory. The increase in interface defects increases the number of traps and the resistance at an interface leading to recombination and reduction in the cell's performance. Two interfaces (ETL/absorber and absorber/HTL) form when the absorber comes into contact with the corresponding CTLs. In order to analyse the effect at interfaces, defect density is increased from 10^10^ to 10^16^ for both ETL/absorber interface and absorber/HTL interface as shown in figures 20 and 21. It has been observed for absorber/ETL that the increase in defect density results in the decrease of *V*_oc_ leading to a drop in PCE. The reduction in *V*_oc_ is because charge carriers (holes) recombine through increasing number of traps states as a result of an increase in defect density. The interface defect density can be controlled by reduction in crystallographic and surface defects at interfaces through fabrication processes [54].

### 3.5. Effect of temperature

Solar cell performance parameters such as *V*_oc_ and *J*_sc_ are dependent on temperature. To analyse the effect temperature of cells is varied from 280 K to 400 K. The effect of temperature on *J*_sc_ and *V*_oc_ can be related through the following equation:

3.2 $$V_{\mathrm{oc}}=\frac{nKT}{q}\log\left(\frac{J_{\mathrm{sc}}}{J_{o}}+1\right).$$

**Figure 22.**
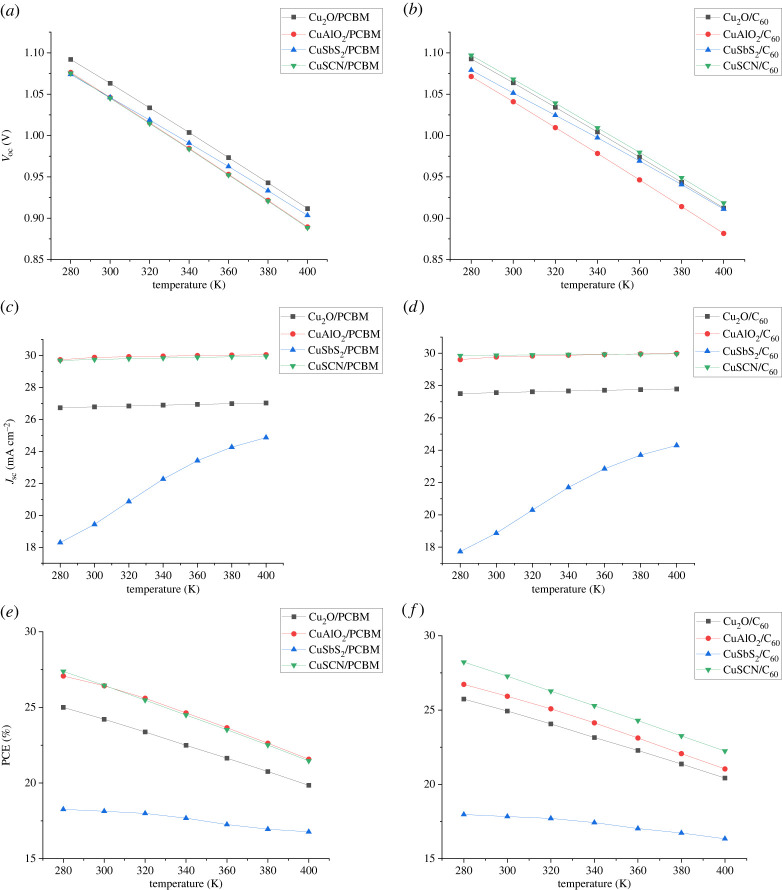
Effect of temperature variation on (*a,b*) *V*_oc_, (*c,d*) *J*_sc_ and (*e,f*) PCE.

Figure 22 shows that with the increase in temperature reverse saturation current increases exponentially which leads to the decrease in *V*_oc_. As can be seen in figure 22 *J*_sc_ also slightly increases with temperature, which is attributed to the decrease in band gap with temperature and thus more absorption leads to an increase in *J*_sc_ [58,59]. The dependence of *E_g_* on temperature in the semiconductor is described by Varshni's relation described in equation (3.3). Where *E_g_*(0) is the value of band gap at zero Kelvin temperature, *α* and *β* are material constants and *T* is temperature.

3.3 $$E_{g}(T)\;=\;E_{g}(0)\;-\frac{\alpha T^{2}}{\left(T+\beta \right)}.$$

PCE degradation with temperature is because of an increase in recombination mainly due to a decrease in *V*_oc_.

### 3.6. Effect of back contact metal electrodes

To collect the electrons by yielding built-in voltage across the solar cell, it is required for a cathode to have a proper work function. To analyse the effect of back contact on performance back, the contact work function is varied from 3.4 eV to 5 eV. Work function affects *V*_oc_ and PCE but it does not affect *J*_sc._

**Figure 23.**
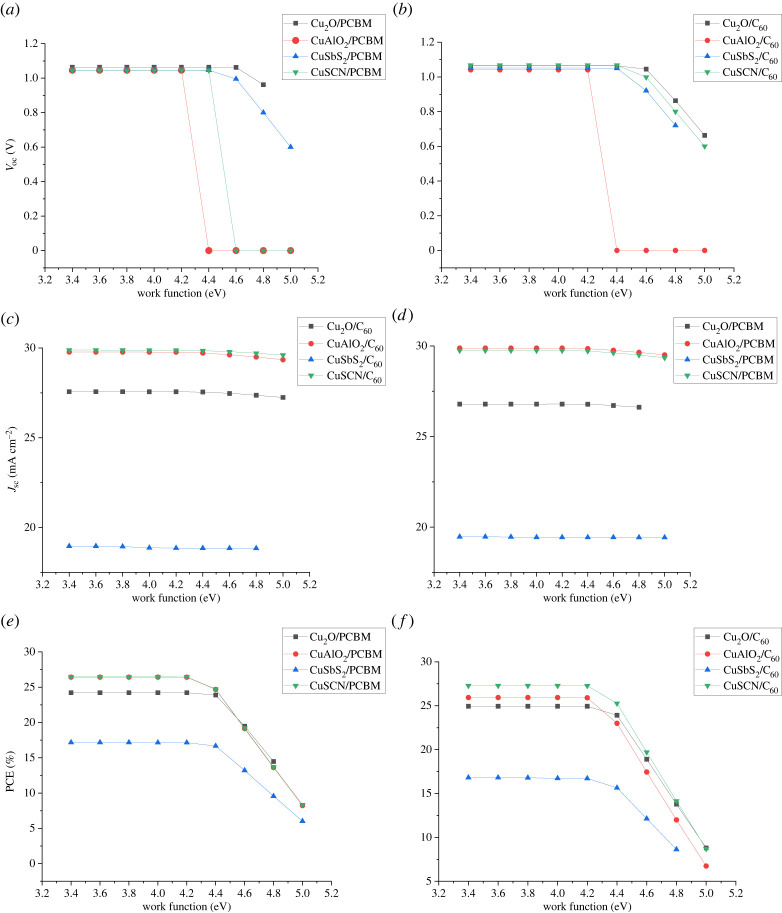
Effect of back contact work function on (*a,b*) *V*_oc_, (*c,d*) *J*_sc_ and (*e,f*) PCE.

Figure 23 shows that *V*_oc_, PCE and FF decrease for higher work function values, however there is no change in current. It has been observed that electrodes with low work function (3.4 eV to 4.2 eV) are suitable for FASnI_3_-based cells because of the low band gap (*E_g_* = 1.41 eV) of FASnI_3_. Thus, it requires a low value of potential difference for charge transport and extraction across the front and back contact as compared to high band gap absorbers such as MAGeI_3_ (*E_g_*1.93) [29,60]. The decrease in efficiency, FF and *V*_oc_ at higher work functions are attributed to the increase in ohmic resistance at the metal and semiconductor interface [61,62].

### 3.7. Effect of reflectance at back contact

**Figure 24.**
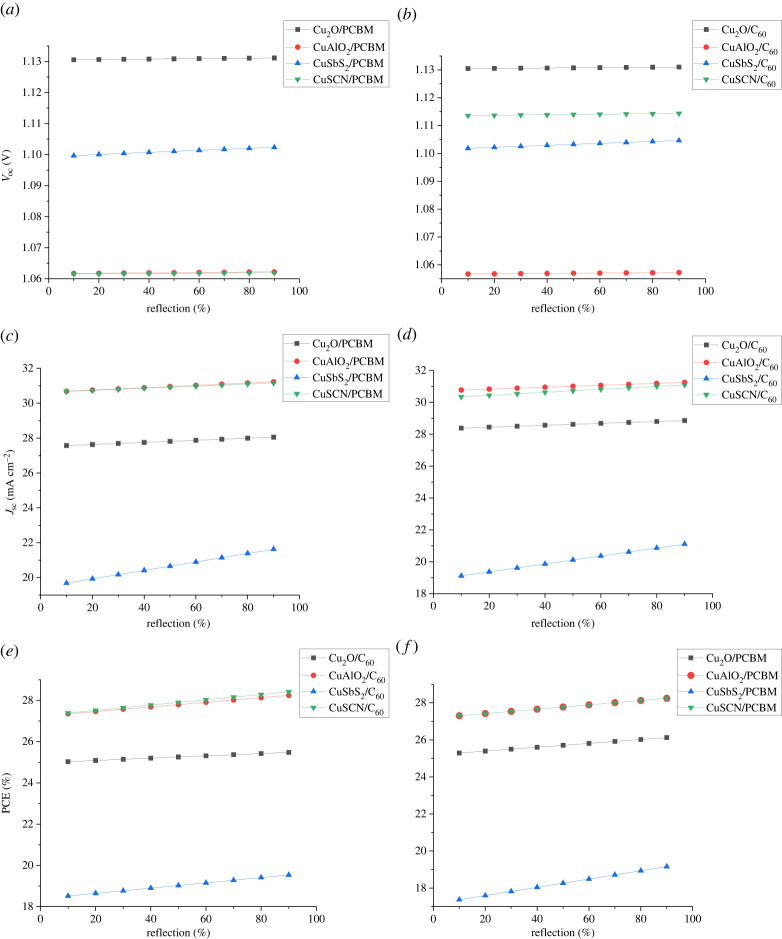
Effect of reflection coating on (*a,b*) *V*_oc_, (*c,d*) *J*_sc_ and (*e,f*) PCE.

Owing to the limitation of absorber thickness in solar cells, photons of larger wavelengths pass through the absorber layer without being absorbed. Reflection coatings on the back side increase the reflectance of the unabsorbed photons of larger wavelengths towards the absorber, thus increasing absorption probability [63]. The reflectance at back contact can be increased by using highly reflective metal with a good surface finish [64]. White paint is also used at back contact sometimes to improve reflectance [65]. To see the effect of back reflectance is varied from 10 to 95%. Figure 24 shows that the back reflection has a vital effect on the *J*_sc_ because no more photons absorber results in more photocurrent generation.

### 3.8. Comparison of performance with conventional nip architecture

**Figure 25.**
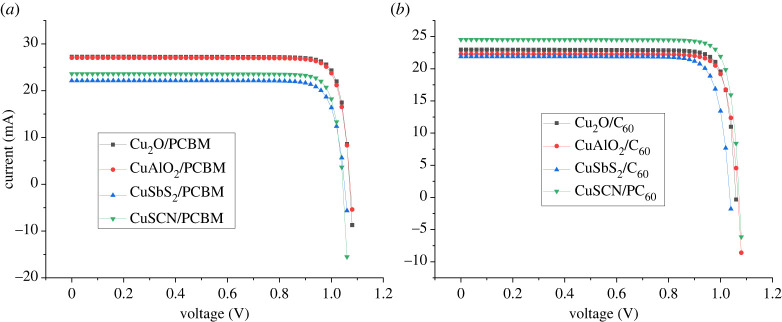
I–V curves of conventional nip architecture.

To analyse the performance of ETLs and HTLs, a simulation was carried out for conventional nip architecture combinations using the optimized parameters mentioned in table 8. When comparing the results of inverted and conventional architecture it can be seen clearly that copper-based HTLs perform better in inverted combination except for CuSbS_2_-based cells which perform better in conventional structures. The reason CuSbS_2_ shows better results for conventional structures is because of its smaller band gap of 1.58 eV than the front ETL layers (PCBM and C_60_). The I–V curves for the conventional cells are shown in figure 25 and table 9 shows the performance.

**Table 9.** Performance parameters of conventional nip architecture.

cells	*V*_oc_	*J*_sc_	FF	PCE %
ITO/PCBM/FASnI_3_/Cu_2_O/Al	1.071129	27.23865	86.3444	25.192
ITO/PCBM/FASnI_3_/CuAlO_2_/Al	1.073057	27.03932	85.7349	24.8758
ITO/PCBM/FASnI_3_/CuSbS_2_/Al	1.051022	22.1453	84.4064	19.6458
ITO/PCBM/FASnI_3_/CuSCN/Al	1.044683	23.60378	86.4641	21.3207
ITO/C_60_/FASnI_3_/Cu_2_O/Al	1.059531	22.96424	86.181	20.969
ITO/C_60_//FASnI_3_/CuAlO_2_/Al	1.06791	22.28187	85.6399	20.378
ITO/C_60_/FASnI_3_/CuSbS_2_/Al	1.036739	21.87101	84.2416	19.1014
ITO/C_60_/FASnI_3_/CuSCN/Al	1.072667	24.53252	86.2043	22.6848

### 3.9. Recent performance of FASnI_3_

The recent developments in FASnI_3_ PSC based on highest practical efficiencies reported over the years are listed in table 10. The first successful tin-based perovskite solar cell was made with an efficiency of 2.1%. It was reported using Spiro-OMeTAD as HTL and TiO_2_ as ETL using SnF_2_ as an additive to improve stability [11]. The following year 5.59% efficiency was obtained for ITO/PVK/PEDOT:PSS/C60/BCP/Ag using different concentrations of SnF_2_ [66]. Further change in structure and architecture helped to further boost the efficiency of Sn-PSC. The highest efficiency uptil 2022 is reported to be 13.82% for ITO/PEDOT:PSS/PER-grad/C_60_/BCP/Ag. The sn-based PSC still has a lot of room for improvement. With the development in research and technology, this improvement can be achieved. This study shows that the Sn-PSC has the potential to surpass 20% PCE.

**Table 10.** Performance of best PCE achieved by FASnI_3_ up until 2020.

device architecture	PCE (%)	year	references
TiO_2_/PVK + 20 mol%SnF_2_/spiro-OMeTAD/Au	2.1	2015	[11]
$\begin{array}{c} \;\mathrm{ITO}/\mathrm{PEDOT}:\mathrm{PSS}/\;\\ \;\mathrm{PVK}+\;10\;\mathrm{mol}\%;\mathrm{Sn}F_{2}/C60/\mathrm{BCP}/\mathrm{Ag}\; \end{array}$	5.59	2016	[66]
$\begin{array}{c} \mathrm{FTO}/c-{\mathrm{TiO}}_{2}/m-\\ {\mathrm{TiO}}_{2}/[\mathrm{PVK}+10\;\mathrm{mol}\%;\;\mathrm{en}/\mathrm{PTAA}/\mathrm{Au} \end{array}$	7.14	2017	[67]
$\begin{array}{c} \;\mathrm{ITO}/\mathrm{PEDOT}:\mathrm{PSS}/ \end{array}\mathrm{PVK}(\pi$-conjugated)/C_60_/BCP/Ag	10.1	2019	[68]
ITO/PEDOT:PSS/PVK+50% LFA/C_60_/BCP/Ag	10.37	2020	[69]
ITO/PEDOT:PSS/PVK-PNCs/C_60_/BCP/Ag	11.39%	2021	[70]
ITO/PEDOT:PSS/PVK-grad/C_60_/Ag	13.82%	2022	[20]

## 4. Conclusion

In this study, eight inverted p-i-n novel FASnI_3_-based solar cells were numerically modelled, optimized and studied in detail. The effect of thickness, doping, defect density, temperature, interface defects, work function and the reflective coating on the performance parameters of solar cells is explained in detail. Recombination electric potential band alignment is explained in detail for absorber/HTL and ETL/absorber interfaces. QE and absorption curves are related to the band gap of HTL. It has been concluded that the performance of solar cells increases with the absorber thickness up to a certain point (optimized value) after which saturation is achieved and then it declines. Most of the cells were optimized for different thickness values ranging from 350 to 1000 nm. Moreover, it has been observed that ETL thickness has a negligible effect on the performance of the solar cell. ETL thickness is kept at 100 nm for all cells. Absorber doping for all cells optimization is achieved at 10^16^ as for most cells the performance decreases at high doping due to an increase in impurity scattering. As for CTLs, doping performance of all the cells increased because of an increase in majority charge carriers. Absorber defects and interface decrease cell performance drastically. From optimization, the best performance for Group 1 was achieved by ITO/CuSCN/FASnI_3_/C_60_/Al with PCE of 27.26% and for Group 2 the best efficiency was achieved by (ITO/CuAlO_2_/FASnI_3_/PCBM/Al) with efficiency of 27.16% due to highly conductive copper-based HTLs and perfect band alignment with FASnI_3_ and high electric potential values at interfaces. It is concluded that in p-i-n inverted cells, the HTLs with larger band gaps are preferred to achieve transparency at the front. All copper-based materials proved to be promising candidates and showed higher electric potential and less recombination at absorber/HTL interfaces except for CuSbS_2_. CuSbS_2_, having a smaller *E_g_* of 1.58 than both ETLs (C_60_ and PCBM), behaves better in the conventional structure. Furthermore, the effect of back contact work function is studied and it is concluded that the metals with work functions lower than 4.2 are suitable for these cells for the reason that aluminum was considered a suitable choice. As temperature increases, the solar cell performance deteriorates, and for reflection coating the performance of the solar cell increases.

## Data Availability

Data available from the Dryad Digital Repository: http://dx.doi.org/10.5061/dryad.4j0zpc8h9 [71].
